# B7-H1 Blockade Increases Survival of Dysfunctional CD8^+^ T Cells and Confers Protection against *Leishmania donovani* Infections

**DOI:** 10.1371/journal.ppat.1000431

**Published:** 2009-05-15

**Authors:** Trupti Joshi, Susana Rodriguez, Vladimir Perovic, Ian A. Cockburn, Simona Stäger

**Affiliations:** 1 Department of Pharmacology and Molecular Sciences, The Johns Hopkins University School of Medicine, Baltimore, Maryland, United States of America; 2 Department of Molecular Microbiology and Immunology, Malaria Research Institute, Johns Hopkins Bloomberg School of Public Health, Baltimore, Maryland, United States of America; University of Wisconsin-Madison, United States of America

## Abstract

Experimental visceral leishmaniasis (VL) represents an exquisite model to study CD8^+^ T cell responses in a context of chronic inflammation and antigen persistence, since it is characterized by chronic infection in the spleen and CD8^+^ T cells are required for the development of protective immunity. However, antigen-specific CD8^+^ T cell responses in VL have so far not been studied, due to the absence of any defined Leishmania-specific CD8^+^ T cell epitopes. In this study, transgenic *Leishmania donovani* parasites expressing ovalbumin were used to characterize the development, function, and fate of Leishmania-specific CD8^+^ T cell responses. Here we show that *L. donovani* parasites evade CD8^+^ T cell responses by limiting their expansion and inducing functional exhaustion and cell death. Dysfunctional CD8^+^ T cells could be partially rescued by in vivo B7-H1 blockade, which increased CD8^+^ T cell survival but failed to restore cytokine production. Nevertheless, B7-H1 blockade significantly reduced the splenic parasite burden. These findings could be exploited for the design of new strategies for immunotherapeutic interventions against VL.

## Author Summary

The protozoan parasite *Leishmania donovani* is the cause of visceral leishmaniasis, a chronic disease that currently affects 12 million people worldwide. We are interested in understanding the immune mechanisms that can control infection. Preliminary studies suggested that CD8^+^ T cells can kill parasites and limit disease; however, studying these important killer cells has been hindered, because we do not know what parasite molecules they recognize. To overcome this, we engineered parasites to express ovalbumin. Since many tools exist to track and measure immune cells targeted at ovalbumin, we can now track the specific CD8^+^ T cell responses that develop upon infection with Leishmania. We found that Leishmania initially induced CD8^+^ T cells to divide and produce molecules such as IFN-gamma that may help them to kill parasites. However, the CD8^+^ T cells rapidly lost their effector function and died off as infection progressed. More encouragingly, though, we were able to recover some CD8^+^ T cell function by blocking immune inhibitory molecules that are induced by parasite infection. The recovered T cells killed parasites and controlled infection. These results are important as they could be exploited for the design of new therapeutic vaccine strategies aimed at inducing protective CD8^+^ T cells.

## Introduction

Antigen-specific CD8^+^ T cell responses are essential for protection and clearance of many microbial pathogens. CD8^+^ T cells recognize peptides which are presented in the context of major histocompatibility complex (MHC) class I via T cell receptor (TCR). Rare naïve CD8^+^ T cells are activated in secondary lymphoid tissues following encounter with dendritic cells expressing peptide/MHCI complexes [1]. Once activated, antigen-specific T cells typically undergo massive expansion, differentiate into effector cells, and acquire the capacity to kill and produce cytokines [2]–[5]. The magnitude of expansion largely depends on the amount of antigen and/or the number of the naïve precursors [6],[7]. This robust proliferation is then followed by a programmed contraction, which occurs independently of duration of infection, magnitude of expansion or antigen dose [7]. Only 5–10% of the cells present during the peak phase survive the contraction, becoming long-lived memory cells [8]. Memory cells show increased responsiveness and undergo dramatic clonal expansion after reencounter with the same antigen, and thereby confer protection [4],[9].

This paradigm of T cell differentiation and memory formation has been mainly derived from models of acute viral and bacterial infections, such as Lymphocytic Choriomeningitis Virus (LCMV; Armstrong strain), Vaccinia Virus and Listeria monocytogenes [2], [7], [10]–[12]. Yet it may not apply to CD8^+^ T cell responses generated in the presence of persistent antigen stimulation. Indeed, several degrees of dysfunction, such as delays in expansion and contraction, anergy, and suppression and exhaustion of effector responses, have been observed during chronic diseases [13]–[18]. The inhibitory receptor PD-1 and its ligand B7-H1 have been shown to play an important role in the regulation of CD8^+^ T cell function in anti-tumour and anti-microbial immunity, and also in the early CD8^+^ T cell fate decisions [19]–[22]. This pathway appears to induce T cell apoptosis and inhibits proliferation and cytokine production upon TCR engagement in vitro [23],[24]. In vivo, B7-H1/PD-1 interaction was shown to control the initiation and reversion of anergy, to inhibit T cell functions, and to be the key pathway in the induction of exhaustion [21],[25],[26]. This functionally inactivated phenotype has also been described in humans, and shown to be reverted by treatment with blocking antibodies to B7-H1, thereby restoring the capacity of CD8^+^ T cells to control disease and decrease viral load [21].

Experimental visceral leishmaniasis (VL) represents an exquisite model to study CD8^+^ T cell responses in a context of chronic inflammation and antigen persistence. In mice, the two main target organs of this disease are the liver and the spleen [27]. While in the liver the infection is self-resolving due to the development of a TH1-dominated granulomatous response, spleens infected with *Leishmania donovani*, the causative agent of visceral leishmaniasis (VL), stay chronically infected. Together with CD4^+^ T cells, CD8^+^ T cells have been shown to be essential for the control of primary infections in various experimental models of Leishmaniasis [28]–[31]. They also appear to be the main mediators of resistance to rechallenge and the major correlates of protection in vaccine-induced immunity against several *Leishmania* species [30], [32]–[35]. However, the onset of these responses seems to be delayed: polyclonal CD8^+^ T cell responses are only detectable 3–4 weeks into the infection in both *L. major* and *L. donovani* infected mice [29],[30]. Due to a lack of knowledge of Leishmania-specific CD8^+^ T cell epitopes, antigen-specific CD8^+^ T cell responses in VL have thus far not been studied.

In this study, transgenic *L. donovani* parasites expressing ovalbumin [36] were used to characterize the development, function and fate of Leishmania-specific CD8^+^ T cell responses during the course of infection. We show that *L. donovani* parasites evade CD8^+^ T cell responses by limiting their expansion and inducing functional exhaustion and cell death.

## Results

### Expansion, duration, and contraction of OT-I CD8^+^T cell responses during *Leishmania donovani* infection

**Figure 1:**
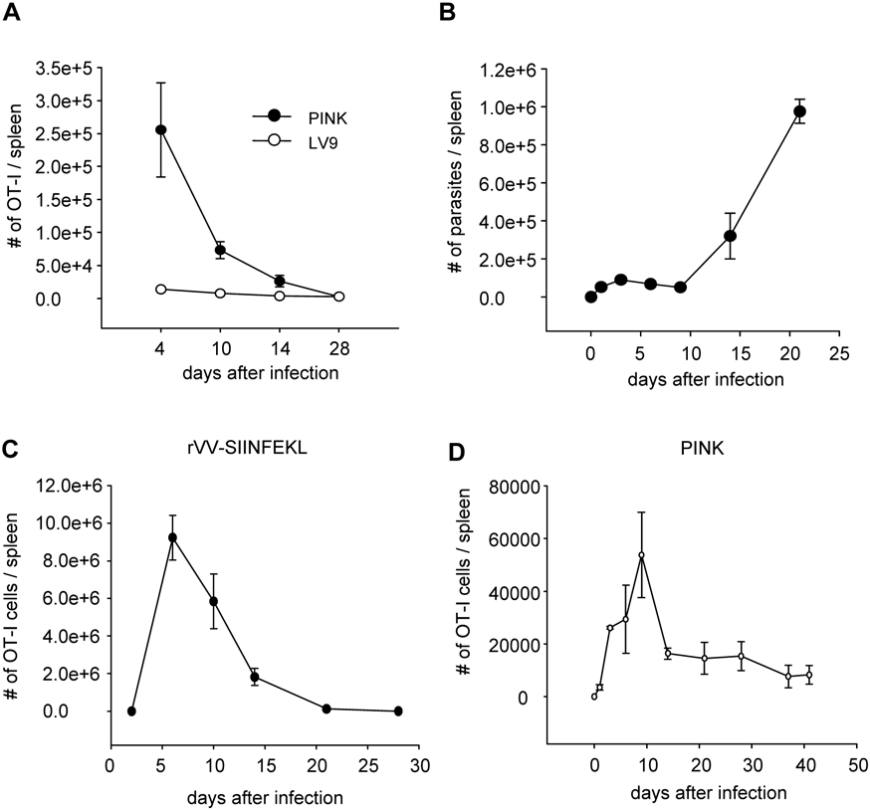
*L. donovani* induces limited expansion of CD8^+^ T cell. (A) 10^5^ OT-I CD8^+^ T cells were adoptively transferred into congenic mice prior to infection with either wild type (LV9) or ovalbumin-transgenic (PINK) *L. donovani* amastigotes. Graph represents the average number±se of OT-I CD8^+^ T cells found in the spleen of each individual mouse during the course of infection. (B) Congenic mice received 10^4^ OT-I CD8^+^ T cells prior to infection with 2×10^7^ PINK amastigotes. Graph represents the average number±se of parasites found in the spleen of each individual mouse. Parasite load was determined by limiting dilutions. (C) The same mice were sacrificed at different time points during infection and OT-I CD8^+^ T cells present in the spleen were enumerated by gating on Ly5.2^+^ CD8^+^ cells. Graph represents the average number±se of cells found in the spleen of each individual mouse during the first 41 days of infection. (D) Congenic mice were infected with rVV-SIINFEKL (10^6^ PFU) i.v. the day after receiving 10^4^ OT-I CD8^+^ T cells. Graph represents the average number±se of Ly5.2^+^ CD8^+^ cells found in the spleen of each individual mouse. All graphs show results representative of 2–3 independent experiments; for all experiments, n = 3.

To determine the extent and significance of bystander activation and distinguish it from antigen-specific responses, we first compared the expansion of adoptively transferred OT-I CD8^+^ T cells in mice infected with wild type (LV9) and Ovalbumin-transgenic (PINK) *Leishmania donovani* parasites. In order to visualize and analyze OT-I CD8^+^ T cell responses in LV9 infected mice, it was necessary to transfer 10^5^ OT-I CD8^+^ T cells per mouse. Although at day 4 after infection there were twice as many OT-I CD8^+^ T cells in the spleen of LV9 infected compared to naive mice, these cells were 20 times fewer than those detected in PINK infected mice (Figure 1A), despite similar splenic parasite burdens (Figure S1). By day 14 the number of OT-I CD8^+^ T cells in PINK infected mice was still 10 times higher then in LV9 infected mice, which had returned to baseline levels. At day 3 p.i., about 30% of the OT-I CD8^+^ T cells in LV9 infected mice had undergone 3–4 rounds of division, downregulated CD62L and expressed high levels of CD44. The percentage of dividing and activated cells remained unchanged throughout the course of infection (data not shown). These data indicate that the proliferative response of OT-I CD8^+^ T cells observed following PINK infection results mainly from antigen-specific stimulation and expansion rather then bystander activation.

We next compared the onset, expansion and dynamic of adoptively transferred OT-I CD8^+^ T cells in PINK infected mice to OT-I CD8^+^ T cell responses induced after infection with recombinant Vaccinia Virus expressing SIINFEKL (rVV-SIINFEKL). Whereas *L. donovani* mounts chronic infections in the spleen (Figure 1B), by contrast, VV is an excellent model for acute viral infections and is mainly cleared by prototypic CD8^+^ T cell response [10]. Adoptive transfer of 10^4^ OT-I CD8^+^ T cells in rVV-SIINFEKL infected mice, resulted in peak expansion at day 6, with 9×10^6^ OT-I CD8^+^ T cells found on average in the spleen. The cells then underwent clonal contraction and by day 21 only about 7% of the cell numbers present during the peak phase were detected (Figure 1C). Following transfer of 10^4^ OT-I CD8^+^ T cells to PINK infected mice, maximum expansion was reached at day 9, and the expansion was 100–200 times lower compared to rVV-SIINFEKL infections. By day 14, the cell number was reduced by 70%. Similar numbers of OT-I CD8^+^ T cells were found in the spleen until day 28 (on average 1.4×10^4^–1.6 10^4^ cells per spleen), after which time the number of OT-I CD8^+^ T cells further decreased, so that by day 41 only 50% of the cells had survived (Figure 1D).

### OT-I CD8 T cells follow a biphasic activation pattern

**Figure 2:**
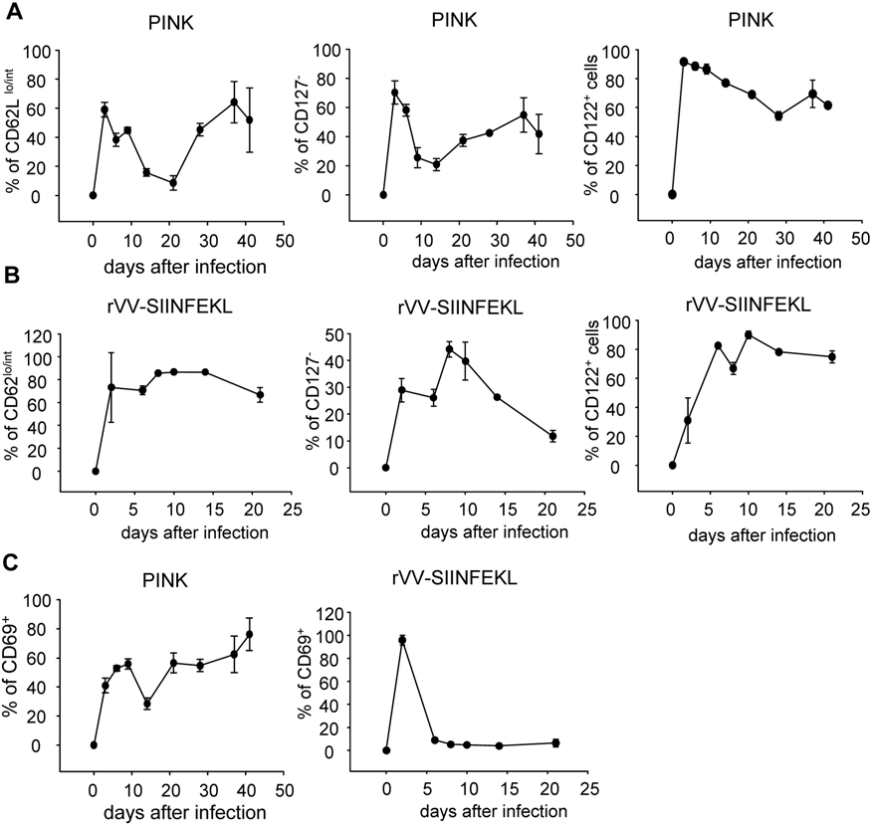
Phenotypic characterization of OT-I CD8^+^ T cell responses in PINK vs. rVV-SIINFEKL infected mice. OT-I CD8^+^ T cells were identified by gating on CD8^+^ Ly5.2^+^ cells. Modulation of cell surface markers in PINK (A) and rVV-SIINFEKL (B) infected mice. (A, B) The percentage of gated cells that expressed low/intermediate levels of CD62L is shown in the left panel; the middle panel indicates the percentage of OT-I CD8^+^ T cells that had downregulated CD127; the right panel represents the percentage of OT-I CD8^+^ T cells positive for CD122. (C) Modulation of CD69 expression in mice infected with PINK (left panel) and rVV-SIINFEKL (right panel). Data represent mean percentages±se and is representative of 2 independent experiments, n = 3.

We next characterized the phenotype of PINK induced OT-I CD8^+^ T cell responses based on the expression of CD62L, CD127, CD44, CD122, and CD69, and compared it to OT-I CD8^+^ T cell responses induced by rVV-SIINFEKL. As shown in Figure 2A, about 40–60% of the OT-I CD8^+^ T cells in mice infected with PINK acquired an effector phenotype by downregulating CD62L during the peak expansion, compared to 90% in rVV-SIINFEKL infected mice (Figure 2B). In the latter group, the majority of the cells remained CD62L^lo/int^ and started to slowly upregulate CD62L only after day 14, suggesting that central memory cells were gradually generated. In contrast, in PINK infected mice 85% of the cells expressed high levels of CD62L at day 14, and at day 21, 92% of the OT-I CD8^+^ T- cells was CD62L^hi^. Between day 21 and 28, cells started down regulating CD62L again and by day 37, 64% of the cells were CD62L^lo/int^, thereby re-acquiring characteristics of an effector phenotype. A similar biphasic pattern of expression was observed with the IL-7R (Figure 2A, central panel). CD127 was down regulated early during the response (day 3–6), but by day 9 about 75% of the cells were CD127^+^. After day 21, cells started down regulating CD127 again and by day 37, more then the half of the cells were CD127 negative. The timing of this shift in the phenotype of the OT-I CD8^+^ T cells corresponds to the expansion in splenic parasite load (Figure 1B).

CD44 was upregulated from day 3 on (Figure S2A). The vast majority of the cells were CD44^hi^ during the first 41 days of infection. Similarly, cells started upregulating CD122 at day 3 and remained CD122^+^ until day 21 (Figure 2A). However, after day 21 about 50% of the cells had downregulated CD122. CD69 has been reported to be transiently expressed during T cell activation and differentiation following antigen-presentation by dendritic cells; however, this molecule has also been shown to be persistently expressed by human and murine T cells in a context of chronic inflammation [37]. CD69 was transiently expressed by all cells present in the spleen of rVV-SIINFEKL infected mice at day 2, while only 3–9% of the cells were CD69^+^ between day 6 and 21 (Figure 2C, right panel). In contrast, in PINK infected mice about 40% of the OT-I CD8^+^ T cells expressed CD69 at day 3. This percentage slightly increased during the course of infection with the exception of day 14, when only 20% of the cells were CD69^+^ (Figure 2C, left panel).

We also monitored the proliferation of OT-1 CD8^+^ T cells by assessing the CFSE dilution over the course of the infection (Figure S3). Between day 2 and 6 after infection, the cells had undergone several rounds of division, resulting in a complete dilution of the CFSE staining (Figure S3). All OT-I CD8^+^ T cells present in the spleen were CFSE^−^ until day 21. In mice infected with PINK, OT-I CD8^+^ T cells had already undergone 4–5 rounds of division at day 3 (Figure S3A), with maximal CFSE dilution observed at day 6. Interestingly, CFSE dilution at day 6 was higher then at days 9, 14, 21, and 28, indicating that the cells present at these later time points had undergone fewer rounds of division then those present at day 6. One possible explanation is that effector cells present in the spleen at day 6 have migrated to the liver, the other site of infection. To test this hypothesis, we enumerated the OT-I CD8^+^ T cells present in the liver during the course of infection (Figure S3B). A maximum of 1500 cells was detected during peak expansion, suggesting that migration of effector cells to the liver was not responsible for the disappearance of these cells from the spleen. This suggests that effector cells that had expanded at day 6 had possibly died and were replaced by newly recruited and activated cells. Notably, these cells did not undergo more then 5–6 rounds of division, even during peak expansion.

Taken together, these results show that OT-I CD8^+^ T cells following PINK infection display a biphasic activation pattern. During the first 9 days of infection, before OT-I CD8^+^ T cells undergo clonal contraction, they exhibit an effector phenotype; this activation results in limited expansion. After the first wave of activation, the majority of the cells that survived clonal contraction were CD62L^hi^ CD44^hi^ CD127^+^ CD122^+^ and KLRG1^−^ (data not shown). This phenotype is similar to that displayed by central memory cells. By week 3 of infection, cells are reactivated, they downregulate CD62L and CD127, but this time they start loosing CD122 expression and their numbers begin to wane.

### *Leishmania donovani* induces mainly polyfunctional CD8^+^ T cells that show signs of exhaustion over the course of infection

**Figure 3:**
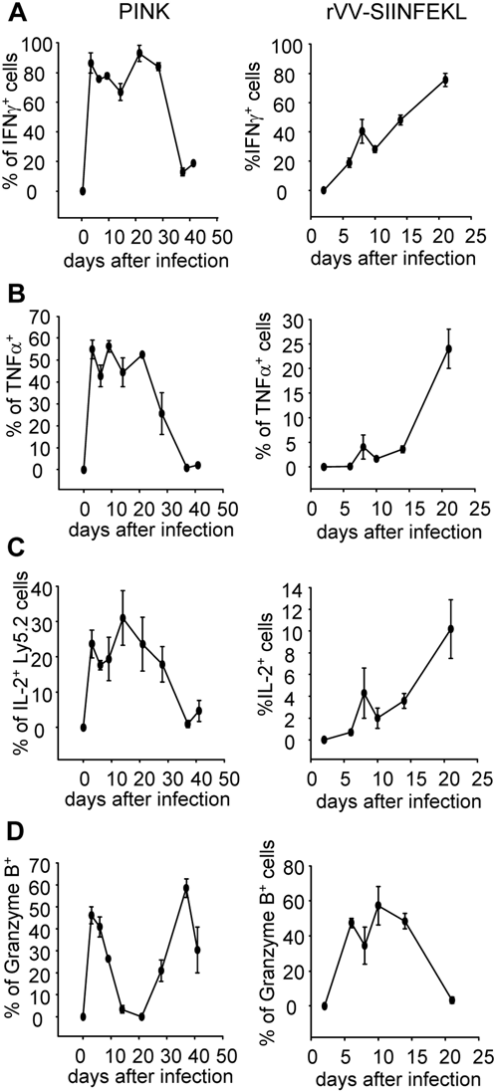
Functional characterization of OT-I CD8^+^ T cell responses in PINK vs. rVV-SIINFEKL infected mice. Splenocytes from PINK (left panels) and rVV-SIINFEKL (right panels) infected mice were restimulated in vitro for 4 h with SIINFEKL peptide and IFNγ, TNFα, IL-2 and Granzyme B were assessed by ICS. Graphs represent the percentage of OT-I CD8^+^ T cells producing IFNγ (A), TNFα (B), IL-2 (C), and granzyme B (D). OT-I CD8^+^ T cells were identified by gating on Ly5.2^+^ cells for the triple ICS staining (IFNγ, TNFα, and IL-2) and on CD8^+^ Ly5.2^+^ for the granzyme B staining. Data represent mean percentages±se, representative of 2 independent experiments, n = 3.

Given the unique alternation of surface phenotype of OT-I CD8^+^ T cell responses during *L. donovani* infections, we were intrigued to investigate the effector function of those cells. After a brief *in vitro* restimulation with the SIINFEKL peptide, cells were stained for INFγ, IL-2, TNFα, Granzyme B, and CD107a. Surprisingly, *L. donovani* induced a very strong CD8^+^ T cell effector response, characterized by a high percentage of cells producing cytokines, of which more then the half were coproducing multiple cytokines (Figure S4A and Figure 3, left panels). 70–90% of OT-I CD8^+^ T cells expressed IFNγ between day 3 and day 28pi in PINK infected mice (Figure 3A, left panel); 45–55% of those cells were concomitantly producing TNFα; and 18–22% were co-producing IL-2 (Figure S4A). These percentages were much greater then those observed following rVV-SIINFEKL infection (Figure 3, right panels). IL-10 was not detected at any time point during infection (data not shown).

In mice infected with rVV-SIINFEKL, we could observe a gradual increase over time in the percentage of polyfunctional cells and in the amount of IFNγ produced per cell (Figure 3, right panels and Figure S4B). This was not the case in PINK infected mice, where CD8^+^ T cell responses became less functional after the first 3–4 weeks of infection. After day 28pi, most of the cells stopped producing IFNγ, and those that did showed a decrease in the mean fluorescence intensity of the staining (Figure S4A). A similar loss of production was observed for TNFα (Figure 3B, left panel) and IL-2 (Figure 3C, left panel): cells secreting these cytokines became progressively less functional from day 14 on.

In rVV-SIINFEKL infected mice, 40–60% of the OT-I CD8^+^ T cells were positive for Granzyme B during the first 2 weeks of infection (Figure 3D). This percentage gradually decreased with the generation of memory cells (Figure 3D, right panel). In contrast, in PINK infected mice the cells displayed a biphasic production pattern: 28–48% of the cells stained positive for Granzyme B during the first 9 days of infection; between day 14 and day 21pi, only 1–2% of the cells were positive (notably, during this period cells display a central memory phenotype); and by day 28pi, cells had reacquired the capacity to produce Granzyme B. We next measured degranulation by cell surface modulation of CD107a (LAMP-1). A pattern of expression similar to that seen for Granzyme B was observed for CD107a (Figure S5).

Thus, following PINK infections OT-I CD8^+^ T cells appear to become dysfunctional over time and express CD69^+^CD44^hi^CD62^lo/int^CD122 ^lo/neg^, however, they maintained degranulation and cytotoxic capacity. These characteristics are very similar to those described for exhausted cells [12],[38]. Interestingly, functional exhaustion of OT-I CD8^+^ T cell was not observed in the liver. In this organ, OT-I CD8^+^ T cells were still producing high levels of IFNγ even at day 41pi (data not shown), suggesting that there might be an organ-specific regulation of CD8^+^ T cell responses and that exhaustion is most likely the consequence of the suppressive splenic environment.

### Ex vivo DCs from infected mice induce a weak proliferation of OT-I CD8^+^ T cells

**Figure 4:**
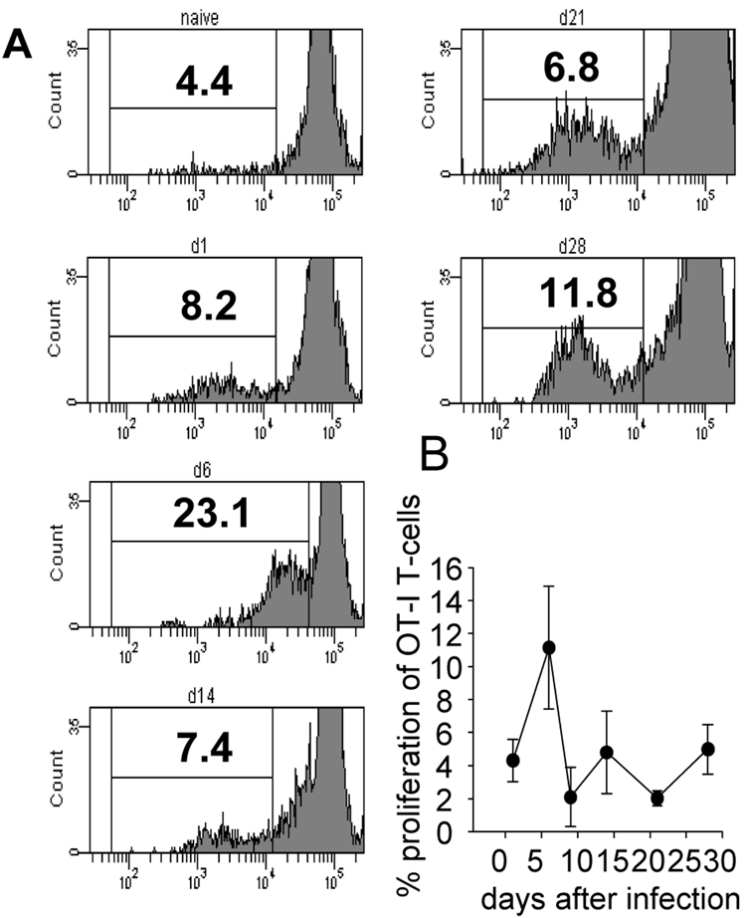
Antigen-presenting capacity of DC purified from PINK infected mice. On various days after infection, MACS enriched CD11c^hi^ DC were coincubated with naïve OT-I CD8^+^ T cells labelled with PKH26. Proliferation of OT-I cells was assessed after 72 h. (A) Representative histograms for several time points of infection are shown. (B) Graph represents the average percentage of proliferation during the course of infection and is representative of 2 independent experiments. For each time point, the percentage background proliferation was calculated after incubating OT-I CD8^+^ T cells with naïve DC and was subsequently subtracted from each value.

In order to understand this biphasic activation pattern, we decided to asses whether the antigen presenting capacity of splenic DC changes during the course of infection. An in vitro proliferation assay using conventional splenic CD11c^hi^ DC purified from infected mice was carried out at different time points of infection. DCs were coincubated with labelled naïve OT-I CD8^+^ T cells for 72 h at 37°C. T cell proliferation was used as a read out for antigen presentation. Maximal proliferation was observed at day 6 after infection: at this time point on average 11.8% of OT-I CD8^+^ T cells had undergone cell division (Figure 4B). In contrast, DC purified at day 9, 14 and 21 induced a poor proliferation of OT-I CD8^+^ T cells (2–3.7%). Their capacity to present antigen increased then again at day 28 (Figure 4B). However, it is important to note that chronic *L. donovani* infections result in splenomegaly. Spleens start to visibly enlarge between day 14 and day 21. As the percentage of DC in the spleen remains the same that means that the capacity of DC to present antigen on a population level at day 21 and 28 is greater then appears from our proliferation assay. Thus it appears that, despite the constant presence of the parasite, antigen presentation by DC during the course of infection follows a biphasic pattern, with peaks at day 6 and day 28pi.

### Conventional CD11c^hi^ DC increasingly express B7-H1 during the course of infection

**Figure 5:**
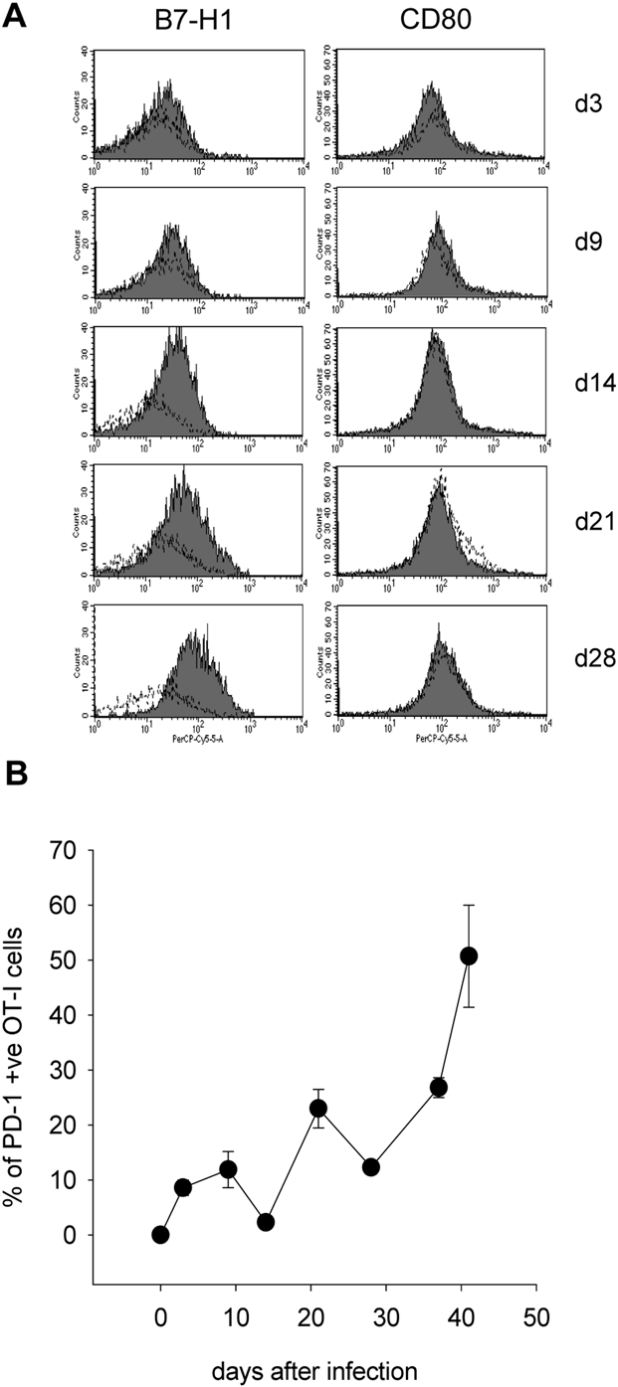
CD11c^hi^ splenic DC upregulate B7-H1 during *L. donovani* infection. (A) B7-H1 (left panels) and CD80 (right panels) expression on CD11c^hi^ splenic DC was assessed on various days after infection. Cells were gated on MHCII^hi^ CD11c^hi^. Dotted lines represent isotyope controls. (B) OT-I CD8^+^ T cells in the spleen of PINK infected mice were identified by gating on Ly5.2^+^ CD8^+^ cells and PD-I expression was assessed at indicated times p.i.. Graph represents mean percentages±se of PD-1 positive cells and show results of 1 of 3 independent experiments, n = 3.

As adoptively transferred OT-I CD8^+^ T cells appeared to acquire characteristics of an exhausted phenotype during chronic infection, we proceeded to examine the B7-H1 expression on dendritic cells from the spleen of infected mice. Conventional CD11c^hi^ dendritic cells significantly and increasingly upregulated B7-H1 during the course of infection (Figure 5A). This upregulation was concomitant with increased PD-1 expression by OT-I CD8^+^ T cells (Figure 5B). These data suggest the potential involvement of the PD-1/B7-H1 pathway in the induction of CD8^+^ T cell exhaustion observed in the spleen of chronically infected mice.

We also investigated the expression of the costimulatory molecules CD40, CD86 and CD80. CD40 and CD86 were increasingly expressed during the course of infection (data not shown); in contrast, CD80 failed to be upregulated (Figure 5A).

### In vivo blockade of B7-H1 rescues OT-I CD8^+^ T cells from cell death and reduces splenic parasite burden

**Figure 6:**
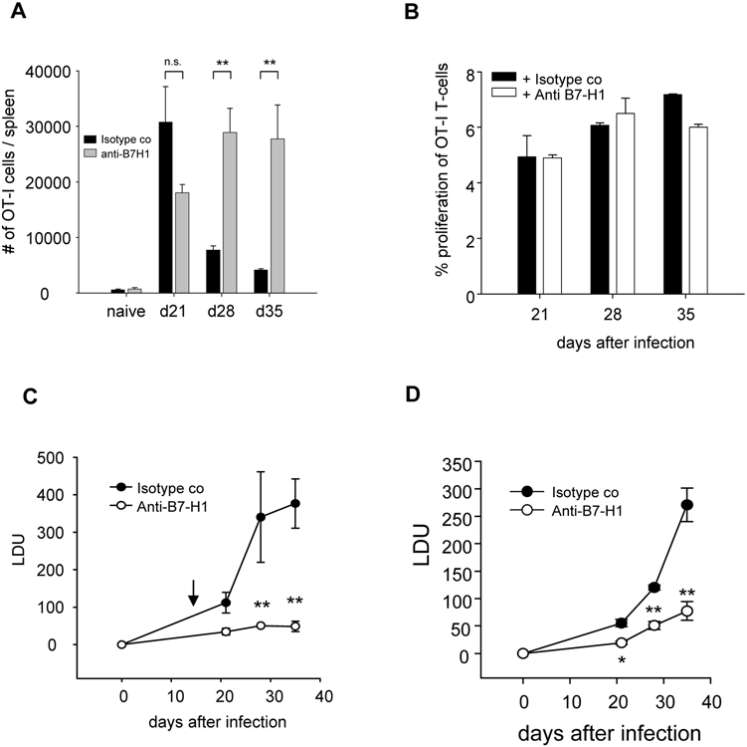
B7-H1 blockade induces protective immunity against *L. donovani*. (A, C) Congenic mice received 10^4^ OT-I CD8^+^ T cells prior to infection with 2×10^7^ PINK amastigotes. From day 15 pi on mice were treated biweekly with anti-B7-H1 antibodies. Animals were sacrificed at indicated times pi. (A) OT-I CD8^+^ T cells were identified by gating CD8^+^ Ly5.2^+^ cells. Graph represents the average numbers of cells per spleen at different time points of infection. (C) Graph represents the splenic parasite burden expressed as Leishman Donovan Units (LDU). All data represent mean±se of one of 2 independent experiments, n = 3. (B) On various days after infection, MACS enriched CD11c^hi^ DC were coincubated with naïve OT-I CD8^+^ T cells labelled with PKH26 in the presence of either 10 µg/ml anti-B7-H1 or an isotype control antibody. Proliferation of OT-I cells was assessed after 72 h. Graph represents the average percentage of proliferation during the course of infection. For each time point, the percentage background proliferation was calculated after incubating OT-I CD8^+^ T cells with naïve DC and was subsequently subtracted from each value. For this experiment, n = 5. (D) C57BL/6 mice were infected with 2×10^7^ LV9 amastigotes and treated biweekly from day 15 pi on with anti-B7-H1 antibodies. Mice were sacrificed at indicated time after infection. Graph represents the splenic parasite burden expressed as LDU. All data represent mean±se of one experiment, n = 5.

To asses whether the PD-1/B7-H1 pathway was involved in the inhibition of cytokine production and/or cell death of adoptively transferred OT-I CD8^+^ T cells, we treated chronically infected mice with anti- B7-H1 blocking antibodies. Treatment started at day 15, when the expression of B7-H1 began to increase; T cell responses and parasite load were monitored. The B7-H1 blockade prevented the dramatic reduction in cell numbers that was observed in untreated mice (Figure 6A). The analysis of cell surface markers of OT-I CD8^+^ T cells in treated versus isotype control group revealed that cells expressing low/intermediate levels of CD62L may be surviving better in treated mice compared to the isotype control group (Figure S6A). In order to investigate whether the PD-1/B7-H1 pathway could be involved in inhibiting the activation of newly migrated naïve OT-I CD8^+^ T cells in the spleen during chronic infection, we tested the capacity of ex-vivo purified dendritic cells to induce proliferation of naïve OT-I CD8^+^ T cells in vitro in the presence or absence of the anti-B7-H1 antibody. B7-H1 blockade did not increase the proliferation of OT-I CD8^+^ T cells (Figure 6B). Taken together, these results imply that the PD-1/B7-H1 pathway could be either involved in the inhibition of the proliferative capacity or in the induction of cell death of effector CD8^+^ T cells. Further investigations are needed in order to clarify this mechanism of action.

Surprisingly, the in vivo blockade only partially restored cytokine production. In fact, IFNγ production was only transiently restored, but OT-I CD8^+^ T cells were still increasingly loosing their capacity to produce IL-2 and TNFα (Figure S6B). No differences were observed in the percentage of cells positive for Granzyme B or in the mean fluorescence intensity of the granzyme B staining.

Despite this functional impairment, mice treated with anti-B7-H1 antibodies were able to control parasite growth, with the splenic parasite burden reduced by 70% at day 21, 85% at day 28, and 87% at day 35 (Figure 6C). These results demonstrate that the PD-1/B7-H1 pathway plays a very important role in the suppression of CD8^+^ T cell responses during chronic *L. donovani* infections.

We next assessed whether B7-H1 blockade would confer protection against infection with the *L. donovani* wild type strain LV9 in a non-transgenic model. We therefore treated C57BL/6 mice chronically infected with LV9 with the anti B7-H1 antibody as previously described and monitored the parasite burden at day 21, 28 and 35 after infection (Figure 6D). B7-H1 blockade significantly reduced the splenic parasite burden at day 21 (65% reduction), 28 (57.5%), and 35 (71.4%), suggesting that endogenous CD8^+^ T cell responses were most likely rescued by the blockade. Interestingly, B7-H1 blockade also conferred protection in the liver, but only at day 21 (53.2% reduction in the parasite burden) and 28 (48.8% reduction), and was ineffective at day 35, when parasite growth in this organ was under control and infection had already significantly decreased (Figure S6C).

### Superinfection at a distant site with rVV-SIINFEKL restores OT-I CD8^+^ T cells responses and reduces parasite burden

**Figure 7:**
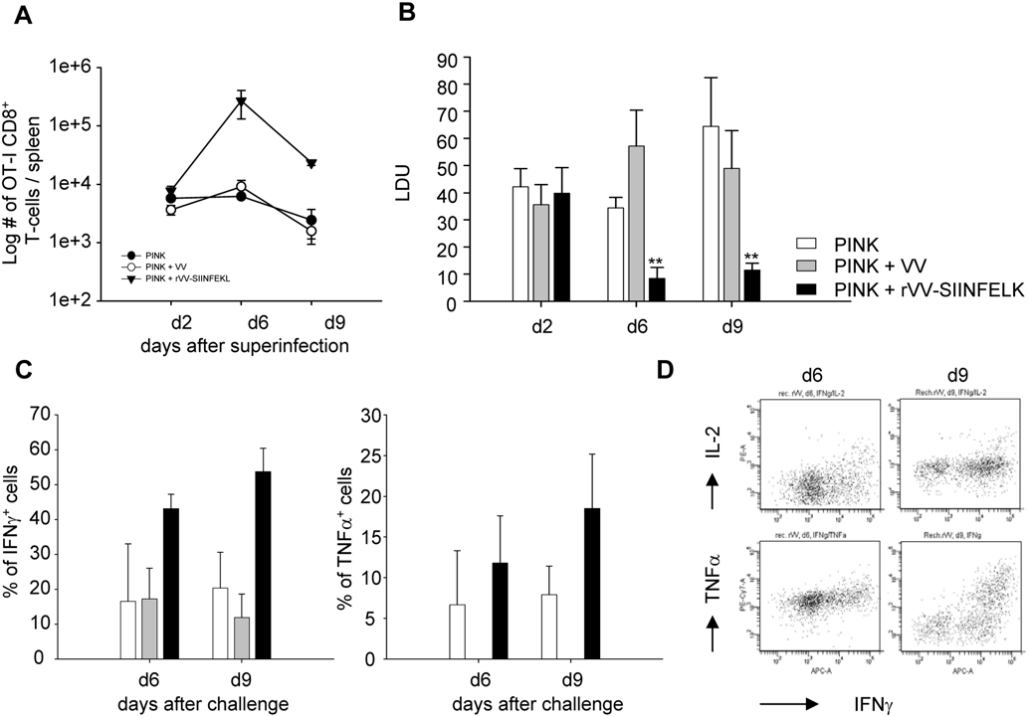
Superinfection with rVV-SIINFEKL, but not with VV, reduces parasite load. 10^4^ OT-I CD8^+^ T cells were adoptively transferred into congenic mice, which were subsequently infected with PINK parasites. At day 32 pi, mice were either untreated or challenged s.c. with VV and/or rVV-SIINFEKL. Mice were sacrificed at indicated times pi. (A) OT-I CD8^+^ T cells present in the spleen were enumerated by gating on Ly5.2^+^ CD8^+^ cells. Graph represents the average number±se of cells found in the spleen of each individual mouse at day 2, 6, and 9 after challenge. (B) The splenic parasite burden expressed as Leishman Donovan Units (LDU) is shown. (C) Splenocytes from the 3 groups of infected mice were restimulated in vitro for 4 h with the SIINFEKL peptide and IFNγ and TNF were assessed by ICS. Graphs represent the percentage of OT-I CD8^+^ T cells producing IFNγ (left panel) and TNFα (right panel). All data represent mean±se, n = 3. (D) Representative plots for the ICS with IFNγ and TNFα at day 6 and 9 after superinfection. All data are representative of 2–3 experiments; n = 3.

To determine whether CD8^+^ T cells were the main mediators of protection following B7-H1 blockade, we induced OT-I CD8^+^ T cell responses by superinfecting chronically infected mice with rVV-SIINFEKL at a distant site. Mice were challenged subcutaneously at day 32 pi with wild type vaccinia virus (VV) or rVV-SIINFEKL, and euthanized 2, 6, and 9 days later. As expected, rVV-SIINFEKL induced a strong proliferation of OT-I CD8^+^ T cells (Figure 7A and Figure S7), whose numbers increased about 40-fold compared to the unchallenged, PINK infected group. Challenge with rVV-SIINFEKL not only induced a proliferative response of OT-I CD8^+^ T cells, but also restored cytokine production (Figure 7C and 7D). Infection with VV did not have any effect on either proliferation or cytokine production, suggesting that the OT-I CD8^+^ T cell response was strictly antigen-specific.

We next compared the splenic parasite burden of mice infected with PINK to that of mice superinfected with VV or rVV-SIINFEKL (Figure 7B). Challenging mice with wild type VV did not alter the course of infection in the spleen. In contrast, infection with rVV-SIINFEKL reduced the parasite burden by 80%. This suggests that reviving CD8^+^ T cell responses during chronic *L. donovani* infections could be a successful strategy for immunotherapeutic interventions.

## Discussion

Our main findings demonstrate that *L. donovani* is able to evade the attack from CD8^+^ T cells by suppressing their expansion and effector function. The data show that despite the constant presence of parasites in the spleen, CD8^+^ T cells responses exhibited a biphasic activation pattern. The first wave of activation led to limited expansion. The second wave resulted in cell death and exhaustion of CD8^+^ T cells. B7-H1 blockade rescued CD8^+^ T cell responses from cell death, but failed to completely restore cytokine production. In spite of this, the parasite burden was considerably reduced after treatment, suggesting that maintenance of effector CD8^+^ T cell responses is crucial for the control of *L. donovani* infections in the spleen.

The adoptive transfer experiments demonstrate for the first time that *L. donovani* induces CD8^+^ T cell responses early during infection. We were able to visualize this early response only because we transferred 10^4^ OT-I CD8^+^ T cells. Physiologically, the number of naturally occurring naïve precursors for a determined epitope is estimated to range between 50 and 1000 cells per mouse [6], [39]–[42]. However, due to the limited expansion capacity of CD8^+^ T cells in this model, transfer of such a low number of cells did not allow us to perform an accurate analysis of endogenous CD8^+^ T cell responses. This might explain why in previous studies the onset of polyclonal responses has been reported to be substantially delayed and could only be detected, mainly in the liver, after 3–4 weeks of infection [30].

Although we transferred the same number of cells with the same epitope specifcity, OT-I CD8^+^ T cells increased in numbers by only 5 fold in Leishmania infected mice, compared to 900 fold in mice infected with rVV-SIINFEKL. In other infection models, expansions up to 50,000-fold were observed [2],[5],[39],[40],[43], suggesting that Leishmania induces a very poor CD8^+^ T cell expansion. When we assessed the antigen-presenting capacity of DC purified from infected animals, we found that even during the early stages of infection, DC were capable of inducing only a weak proliferative response of naïve OT-I CD8^+^ T cells. This is not surprising, since processing of Leishmania antigens for MHCI presentation has been shown to be TAP-and proteosome-independent [44], a pathway that is much less efficient then the conventional ER-based, TAP-dependent pathway for Class I presentation. We also noted that OT-I CD8^+^ T cells present in the spleen at day 9 p.i. had undergone fewer rounds of division then those detected at day 6 p.i., implying that between day 6 and 9 effector cells had died and were replaced with newly activated CD8^+^ T cells. This suggests that expansion could also be limited by cell death of effector cells. We also cannot rule out the possibility of defective recruitment of CD8^+^ T cells into the spleen. Leishmania infections are known to interfere with chemokine expression [45]–[47], including CCL3 [48], a chemokine that was recently shown to be involved in guiding CD8^+^ T cells to sites of CD4^+^ T cell-dendritic cell interaction [49]. Hence, the limited expansion of OT-I CD8^+^ T cells in *L. donovani* infected mice might be due to a combination of several factors, including low antigen load, poor recruitment of CD8^+^ T cells and/or increased cell death of effector cells. Mechanisms responsible for this poor expansion are currently under investigation.

In agreement with the previous literature, OT-I CD8^+^ T cell expansion was followed by contraction at day 14 despite antigen persistence [7]. This contraction was much steeper then in rVV-SIINFEKL infected mice, suggesting that cells were dying more rapidly. One of the most striking findings was that about 80% of the cells that had survived contraction showed a central memory-like phenotype, by expressing CD62L^hi^, CD44^hi^, CD127^+^, CD122^+^, CD69^−^. These cells produced high amounts of IFNγ upon restimulation and the majority were polyfunctional. Additionally, they did not produce Granzyme B. The remaining 20% displayed an effector phenotype (CD62L^lo^, CD127^−^), suggesting that effector memory cells were not generated. A similar population of central memory-like cells has been recently observed in mice infected with *Trypanosoma cruzi* [50]. Despite being capable of antigen-independent survival, this population was shown to be maintained for over a year in the presence of antigen persistence. A recent report suggested a crucial role of T-bet as a molecular switch between central- and effector memory cells [51],[52]. T-bet deficiency was shown to enhance generation of central memory cells. As T-bet is also involved in the induction of enhanced CD122 expression [53], and CD122 expression by OT-I CD8^+^ T cells is gradually decreased during the course of VL, it is possible that this molecule might not be properly induced in Leishmania-specific CD8^+^ T cells.

Another interesting observation was the biphasic activation pattern of OT-I CD8^+^ T cells, which reflects the variation in the capacity of DC to present antigen during the course of infection. This biphasic pattern can be in part explained by the biology of the Leishmania infections. These protozoan parasites are obligate intracellular pathogens that preferentially reside in macrophages, but they can also be found in other cell types [54],[55], including DC [56]. During the first wave of expansion, the majority of the cells capable of cross-presenting Leishmania antigen via MHCI is most likely killed by CTLs so that at the end of contraction very few DC presenting antigen survive and most of the parasites reside in cells that are unable to cross-present antigen. For the second wave of expansion, parasites will have first to be released from those cells in order to be phagocytosed by DC and then killed and processed for antigen presentation to CD8^+^ T cells. This explains why between d14 and d21 p.i. DC showed a very poor antigen presenting capacity. However, the amount of antigen presented during this period, although little, could still be enough to restimulate a memory response. Thus, OT-I CD8^+^ T cell responses might be already impaired at this early stage of infection. Indeed, the second wave of activation did not result in expansion, but in functional exhaustion and cell death of the OT-I CD8^+^ T cells.

This dysfunctional response could be a consequence of an intrinsic problem following defective priming and/or could result from a suppressive splenic environment. Although we can not rule out that OT-I CD8^+^ T cell responses in *L. donovani* infected mice might also have some intrinsic defects, our data support the second scenario. Indeed conventional CD11c^hi^ splenic DC seemed to increasingly express the inhibitory molecule B7-H1 and failed to upregulate the costimulatory molecule CD80. B7-H1 is constitutively expressed on subsets of macrophages, B-cells and thymocytes, and can be induced on dendritic cells, endothelial and epithelial cells [19],[57]. Upregulation of B7-H1 on DC has been observed during several chronic infections and in a wide range of tumors [20],[26],[58],[59]. Our results show that in vivo blockade of B7-H1 during chronic *L. donovani* infection increased the survival of OT-I CD8^+^ T cells. B7-H1 is thought to inhibit T cell proliferation and cytokine production by ligation with the PD-1 receptor [23]. Through ligation with a yet unknown receptor, B7-H1 can also induce programmed cell death of effector T cells [60]. Increased survival of OT-I CD8^+^ T cells after B7-H1 blockade could therefore result from restoration of the proliferative capacity or inhibition of induced cell death of effector CD8^+^ T cells.

In contrast to what has been recently reported in the literature [21],[26], *in vivo* blockade of B7-H1 during chronic VL did not completely restore the functional capacity of exhausted OT-I CD8^+^ T cells. This suggests that suppression of cytokine production by CD8^+^ T cells during *L. donovani* infection might be induced by mechanisms other than through the B7-H1/PD-1 pathway. A recent report has demonstrated a synergistic effect between TGFβ and the B7-H1/PD-1 axis in suppressing CD8^+^ T cell responses [61]. As TGFβ does not seem to play an important role during chronic *L. donovani* infections [62], the possibility that IL-10, which is elevated in both mouse and human VL [63]–[69], could synergistically act with the B7-H1/PD-1 axis, needs to be investigated. To our surprise, B7-H1 blockade resulted in significant decrease in the parasite burden even if it failed to fully restore IFNγ production. While CD4^+^ T cells are clearly an important source of IFNγ in VL, recently we have shown that therapeutic intervention with antigen-specific CD8^+^ T cells in chronically infected mice dramatically reduced the parasite burden [36], indicating that CD8^+^ T cells might play a much more important role than previously thought. The current data reinforce these findings by showing that OT-I CD8^+^ T cells rescued from cell death by blocking B7-H1 or by superinfecting mice with rVV-SIINFEKL resulted in host protection. The mechanism of protection is not clear and might not merely rely on IFNγ production, as only 20% of the OT-I CD8^+^ T cells were producing low amounts of IFNγ at d35 pi. Nonetheless, most of the cells were granzyme B positive and were degranulating upon restimulation, suggesting that they have retained their cytotoxic capacity. To date there is no evidence that CD8^+^ T cells can mediate protection against *L. donovani* through their cytotoxic activity.

In summary, this study shows that restoration of dysfunctional CD8^+^ T cell responses induced by chronic *L. donovani* infections results in disease control and host protection. This implies that targeting CD8^+^ T cell responses by therapeutic vaccination could be beneficial against chronic *L. donovani* infections. Moreover, these findings might provide insights into the development of novel strategies for therapeutic vaccination or other interventions aimed at inducing CD8^+^ T cell responses, which might circumvent and/or neutralize the immunosuppressive environment of the spleen.

## Materials and Methods

### Mice, parasites, and virus

C57BL/6-*Tg(OT-I)-RAG1^tm1Mom^* mice were purchased from Taconic; B6-Ly5.2 congenic mice were obtained from The National Cancer Institute (Frederick, MD, USA), and B6.129S7-*Rag1^tm1Mom^*/J from The Jackson Laboratory. All mice were housed in the Johns Hopkins University animal facilities (Baltimore, MD) under specific pathogen-free conditions and used at 6–8 weeks of age. All experiments were approved by the Animal Care and Use Committee of the Johns Hopkins University School of Medicine.

Ovalbumin-transgenic parasites were a gift from P.Kaye and D.F. Smith (University of York, UK) and were generated as previously described[36]. Wild type and ovalbumin transgenic *Leishmania donovani* (strain LV9) parasites were maintained by serial passage in B6.129S7-*Rag1^tm1Mom^*/J mice, and amastigotes were isolated from the spleens of infected animals. Mice were infected by injecting 2×10^7^ amastigotes intravenously via the lateral tail vein. Hepatic and splenic parasite burdens were determined either by limiting dilutions [31]or by examining methanol-fixed, Giemsa stained tissue impression smears[70]. Data are presented as number of parasites per spleen or as Leishman Donovan Units (LDU).

The recombinant vaccinia virus (rVV) encoding SIINFEKL (chicken ovalbumin 257–264) was a gift from F. Zavala (School of Public Health, JHU, Baltimore) [71]. Mice were infected intravenously or subcutaneously with 2×10^6^ pfu.

### Adoptive transfer of OT-I cells

OT-I/RAG1 mice, transgenic for a T cell receptor specific for chicken ovalbumin 257–264 presented by the MHC class I molecule H-2 Kb, were used as T cell donors. CD8^+^ T cells were enriched from splenocytes of naïve OT-I/RAG1 animals using magnetic cell sorting (MACS), following manufacturers instructions (Miltenyi Biotech). Naïve CD8^+^ T cells were then sorted to >98% purity using FACSVantage (Becton Dickinson) based on their expression of CD44 and CD62L. After sorting, cells were labelled with CFSE. Briefly, cells were resuspended at 5×10^7^/ml in PBS and incubated with 2.5 µg/ml CFSE (Molecular Probes, USA) for 10 min. at 37°C. The reaction was stopped by addition of ice cold RPMI. Samples were then analyzed using a FACSDiva (Becton Dickinson) for CFSE uptake prior to adoptive transfer. Depending on the experiment, 1×10^4^ or 5×10^4^ cells were injected into the lateral tail vein of B6-Ly5.2 congenic mice. Animals were infected the day after with rVV-SIINFEKL and/or with wild type or ovalbumin-transgenic *Leishmania donovani*.

### Superinfection with rVV-SIINFEKL

1×10^4^ sorted naïve OT-I CD8^+^ T cells were adoptively transferred into B6-Ly5.2 congenic mice prior to infection with 2×10^7^ ovalbumin expressing *L. donovani* amastigotes. At day 32pi mice were superinfected subcutaneously at the base of the tail with 2×10^6^ PFU of Vaccinia Virus (VV) or with recombinant VV expressing the SIINFEKL peptide (rVV-SIINFEKL). Animals were sacrificed at d2, d6 and d9 after infection with rVV-SIINFEKL.

### Flow cytometry

OT-I CD8^+^ T cells were identified by staining splenocytes, lymphnode cells and hepatic mononuclear cells with biotinylated anti-CD45.2 antibody followed by PerCP-streptavidin (BD Biosciences). The following antibodies were used to further characterize the OT-I response: APC-conjugated anti-CD44 and anti-CD8, PE-conjugated anti-CD62L, anti-CD69, anti-CD122, anti-CD127 (all obtained from BD Biosciences), and anti-PD-1 (eBioscience). Splenocytes were also stained with APC-conjugated anti-CD11c, FITC-conjugated anti-MHCII, PE-conjugated anti-CD86, PE-Cy5.5 conjugated anti-CD80, and biotinylated anti-B7H1 and anti-CD40, followed by PerCP-conjugated streptavidin (all purchased by BD Biosciences). For all surface markers, cells were directly stained following standard protocols. For intracellular staining, splenocytes were stimulated with the SIINFEKL peptide for 4 hours in the presence of Brefeldin A and then stained with biotinylated anti-CD45.2, followed by PerCP-conjugated strepavidin. After fixation, cells were permeabilized and stained with anti-Granzyme B (Invitrogen) or APC-conjugated anti-INFγ (BD Biosciences), PE-conjugated anti IL-2 (BD Biosciences), and PE-Cy7-conjugated anti-TNFα (eBioscience). Cells were also stained with PE-conjugated anti-CD107 (eBioscience) following the protocol described by Betts et al. [72].

Flowcytometric analysis was performed with a LSRII (Becton Dickinson). One to two millions cells per sample were acquired and analysed with the FACSDiva or with CellQuest software.

### In vitro OT-I proliferation assay

Spleen of naïve and ovalbumin-transgenic *L. donovani* infected mice were digested with 0.4 mg/ml collagenase D for 30 minutes at room temperature. Conventional CD11c^hi^ dendritic cells were then enriched by MACS using CD11c microbeads (purity 80–85%).

Dendritic cells were seeded at a concentration of 2×10^4^ cells/well in a 96 wells plate.

After negative selection with anti-CD11c microbeads, CD8^+^ T cells were purified from the spleen of naïve OT-I/RAG1 mice using magnetic cell sorting (Miltenyi Biotech) (85–90% purity). CD8^+^ OT-I T cells were then labelled using a red fluorescent cell linker PKH26 (Sigma) in order to track proliferation. They were then added to the ex-vivo purified dendritic cells at a concentration of 10^5^/well. 1 ng/ml of recombinant human IL-2 was also added to the wells. The proliferation of OT-I T cells was assessed 72 h later by flowcytometry using FACSDiva (BD Biosciences) and analysed with the FACSDiva software. Results are expressed as percentage of OT-I CD8^+^ T cells that have undergone one or more rounds of division. The percentage of cells that entered division when incubated with DCs from a naive animal was subtracted from this value.

### B7H-1 blockade

Antagonistic mouse B7-H1 monoclonal antibody (clone 10B5) was purified on a protein G column from the supernatant of the hybridoma cell line. The hybridoma cell line was a gift from L.Chen (Johns Hopkins University, School of Medicine, Baltimore). Hamster IgG (Sigma) was used as isotype control. Mice were treated every 4 days with 100 µg of antibody i.p. The first treatment started at day 15 p.i. Before treatment, antibodies were tested for functionally relevant LPS contamination, by assaying their ability to synergize with IFNγ for the induction of inducible NO synthase [73]. No activity was detectable in such assays (sensitivity,1 ng/ml LPS; data not shown).

### Statistical analysis

Results were analyzed using an unpaired Student *t*-test. *P*<0.05 was considered significant. All experiments were repeated at least twice.

## Supporting Information

Figure S1 Comparison of the splenic parasite burden in mice infected with PINK vs. LV9. Parasite numbers were determined by limiting dilutions.
(0.13 MB TIF)

Figure S2 Modulation of expression of cell surface markers CD62L, CD69, CD127, CD122 at indicated times pi. Representative plots for PINK (A) and rVV-SIINFEKL (B) infected mice.
(1.35 MB TIF)

Figure S3 (A) CFSE dilution of OT-I CD8^+^ T cells on various times pi. OT-I CD8^+^ T cells were identified by gating on Ly5.2+ CD8^+^ cells. (B) Average numbers±se of OT-I CD8^+^ T cells found in the liver at different time point of infection.
(0.35 MB TIF)

Figure S4 Cytokine production by adoptively transferred OT-I CD8^+^ T-cells after infection with PINK (A) or rVV-SIINFEKL (B) on various time points pi. Cytokine production was assessed by ICS after 4 h stimulation with the SIINFEKL peptide. Representative plots for IL-2, IFNg, TNFa, and granzyme B stainigs are shown.
(1.37 MB TIF)

Figure S5 On indicated times pi, splenocytes were restimulated for 4 h with the SIINFEKL peptide and CD107a expression on cells was assessed. Representative plots for each time point are shown.
(0.17 MB TIF)

Figure S6 Congenic mice received 104 OT-I CD8^+^ T cells prior to infection with 2×107 PINK amastigotes. From day 15 pi on mice were treated biweekly with anti-B7-H1 antibodies. Animals were sacrificed at indicated times pi. (A) Modulation of CD62L and CD122 expression on OT-I CD8^+^ T cells, identified by gating on Ly5.2+ CD8^+^ cells. Mean percentages of cells expressing low/intermediate levels of CD62L (left panel) and CD122 (right panel) are shown. (B) Splenocytes from ant-B7-H1 treated and isotype control treated mice were restimulated in vitro for 4 h with the SIINFEKL peptide and IFNg and TNFa were assessed by ICS. Graphs represent the percentage of OT-I CD8^+^ T cells producing IFNg (left panel) and TNFa (right panel). All data represent mean±se, n = 3. (C) C57BL/6 mice were infected with 2×107 LV9 amastigotes and treated biweekly from day 15 pi on with anti-B7-H1 antibodies. Mice were sacrificed at indicated time after infection. Graph represents the hepatic parasite burden expressed as LDU. All data represent mean±se of one experiment, n = 5.
(0.28 MB TIF)

Figure S7 Modulation of expression of CD62L, CD69, CD122, and CD127 after superinfection with VV and/or rVV-SIINFEKL. Representative plots for both groups at day 6 and 9 after challenge are shown.
(0.47 MB TIF)
