# Supplementary figures and images for: B7-H1 Blockade Increases Survival of Dysfunctional CD8+ T Cells and Confers Protection against Leishmania donovani Infections
Source: PLoS Pathog. 2009 May 15;5(5):e1000431. doi: 10.1371/journal.ppat.1000431 (PMC2674929; doi:10.1371/journal.ppat.1000431)

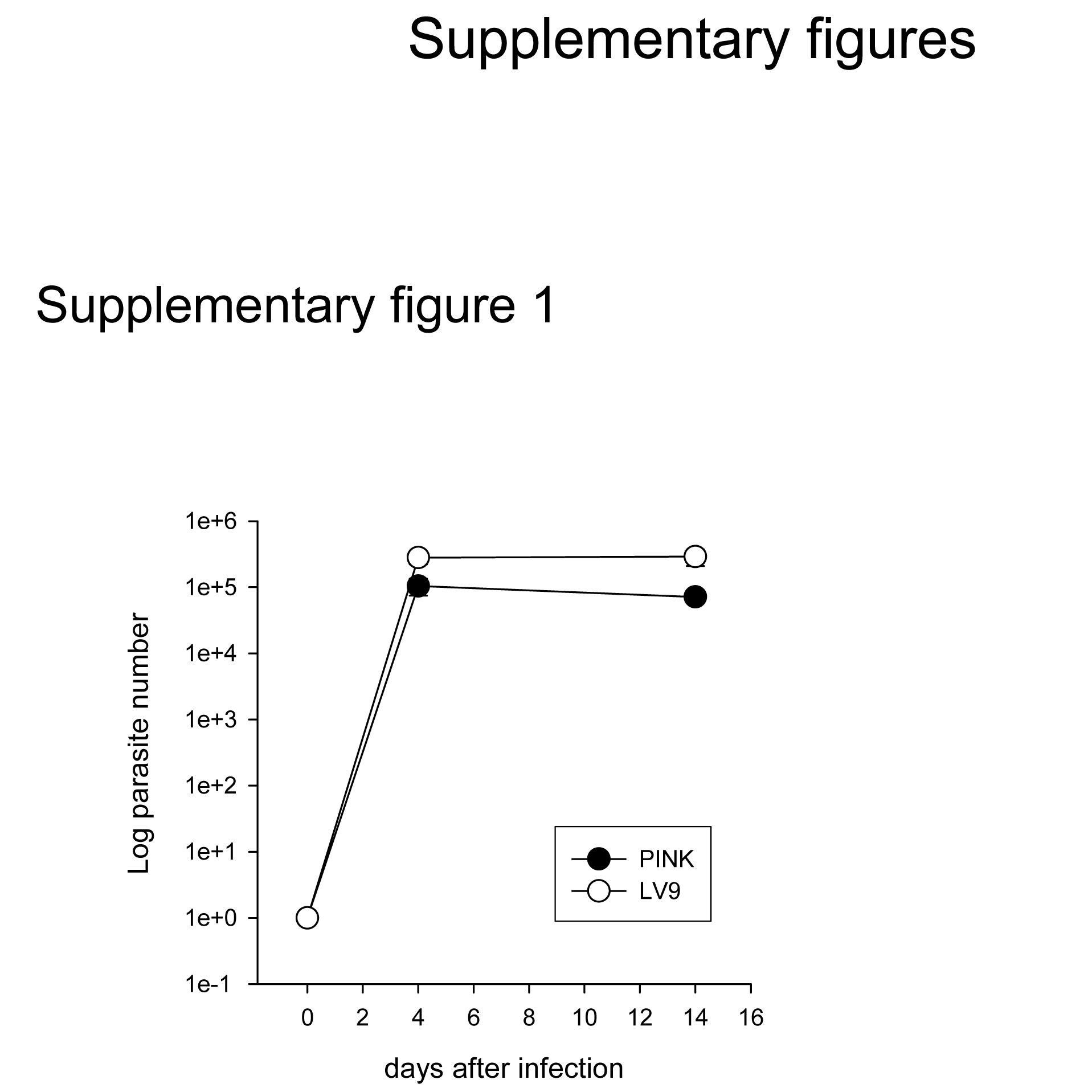

Supplement: Figure S1 — Comparison of the splenic parasite burden in mice infected with PINK vs. LV9. Parasite numbers were determined by limiting dilutions. (0.13 MB TIF) [file ppat.1000431.s001.tif]

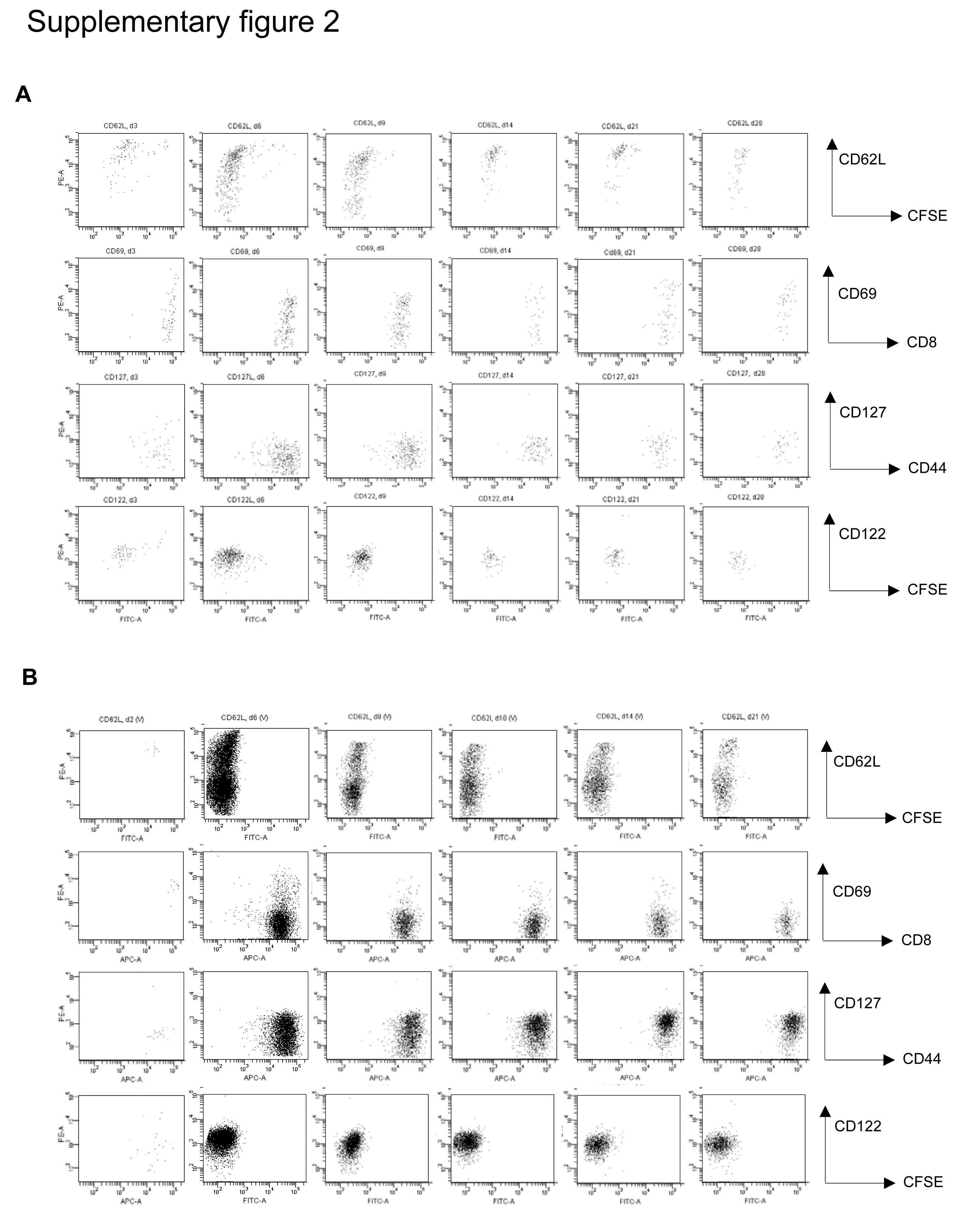

Supplement: Figure S2 — Modulation of expression of cell surface markers CD62L, CD69, CD127, CD122 at indicated times pi. Representative plots for PINK (A) and rVV-SIINFEKL (B) infected mice. (1.35 MB TIF) [file ppat.1000431.s002.tif]

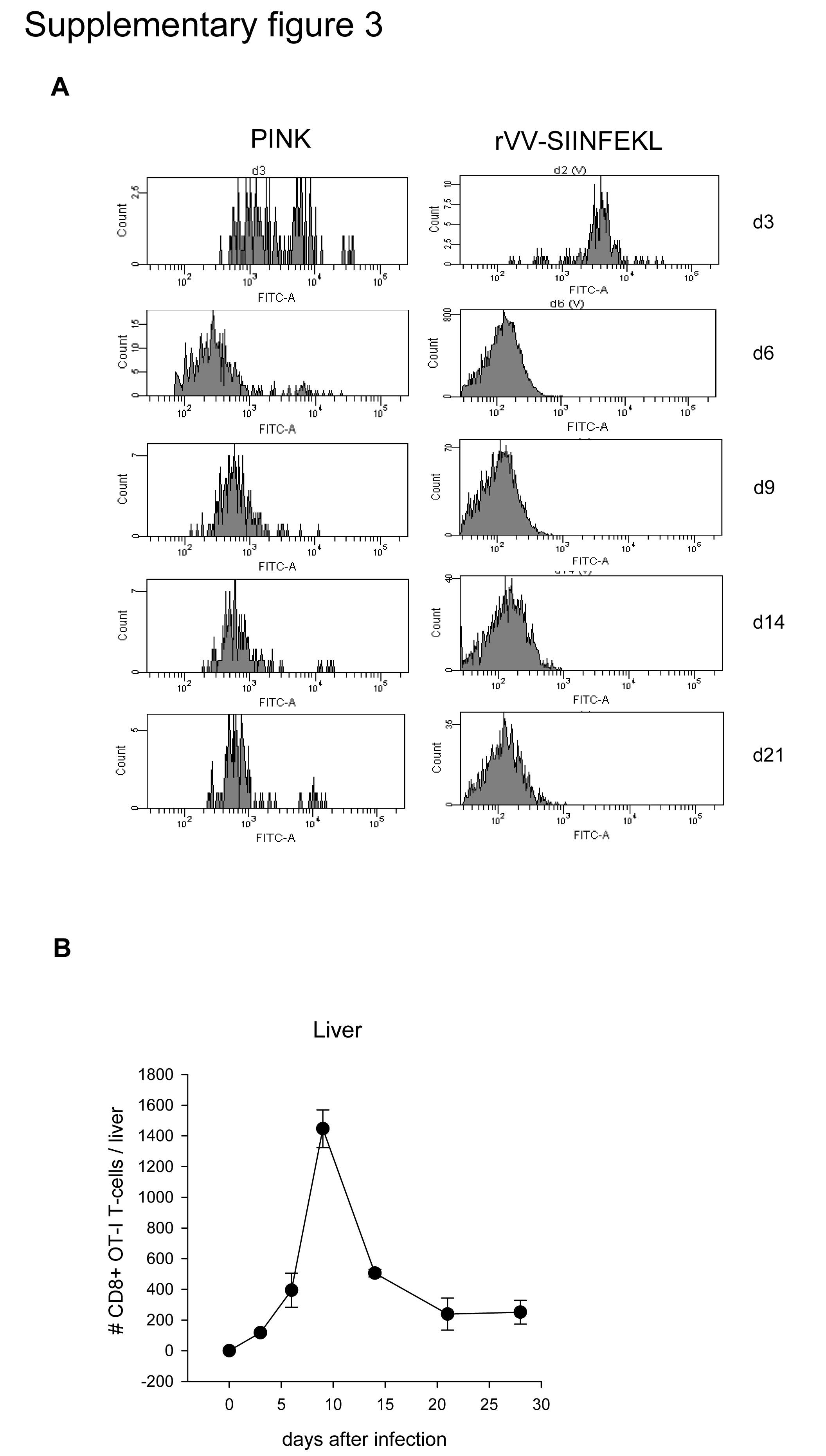

Supplement: Figure S3 — (A) CFSE dilution of OT-I CD8+ T cells on various times pi. OT-I CD8+ T cells were identified by gating on Ly5.2+ CD8+ cells. (B) Average numbers±se of OT-I CD8+ T cells found in the liver at different time point of infection. (0.35 MB TIF) [file ppat.1000431.s003.tif]

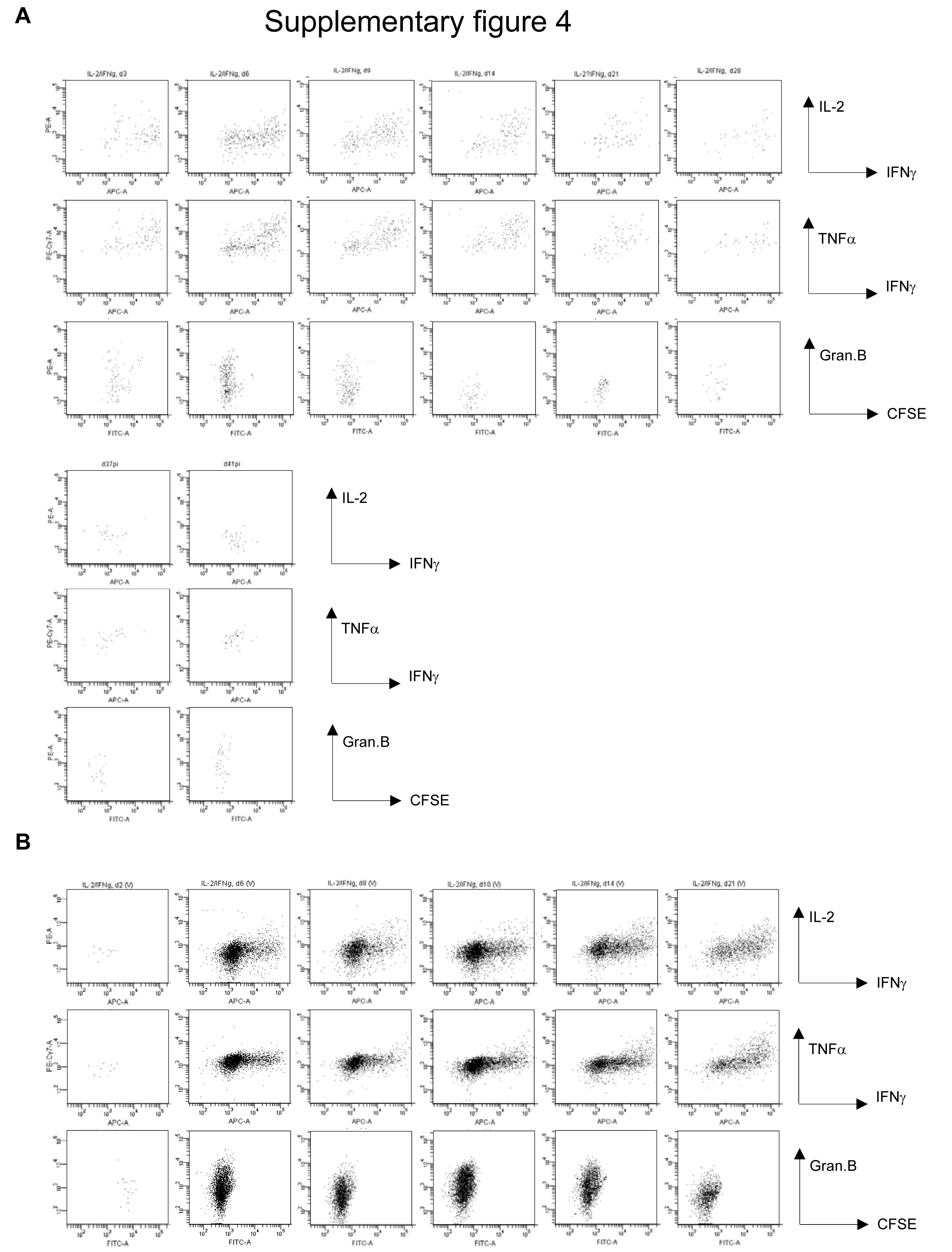

Supplement: Figure S4 — Cytokine production by adoptively transferred OT-I CD8+ T-cells after infection with PINK (A) or rVV-SIINFEKL (B) on various time points pi. Cytokine production was assessed by ICS after 4 h stimulation with the SIINFEKL peptide. Representative plots for IL-2, IFNg, TNFa, and granzyme B stainigs are shown. (1.37 MB TIF) [file ppat.1000431.s004.tif]

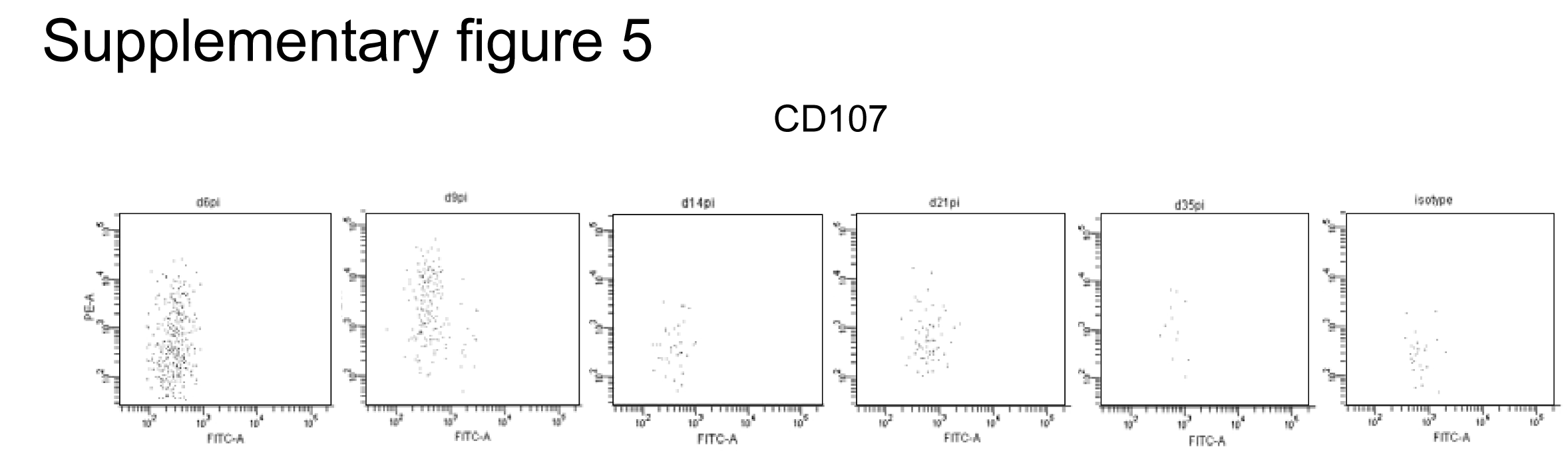

Supplement: Figure S5 — On indicated times pi, splenocytes were restimulated for 4 h with the SIINFEKL peptide and CD107a expression on cells was assessed. Representative plots for each time point are shown. (0.17 MB TIF) [file ppat.1000431.s005.tif]

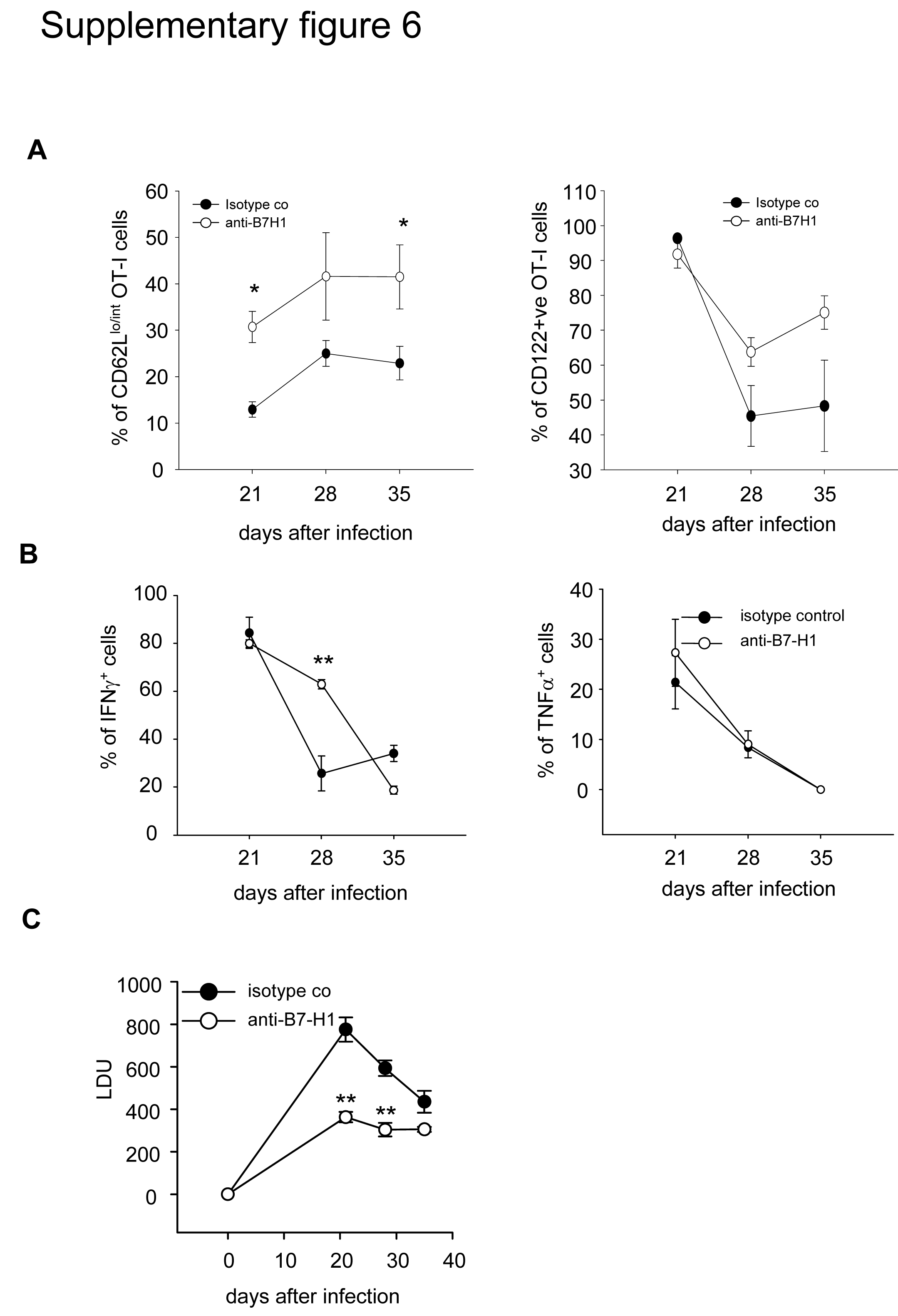

Supplement: Figure S6 — Congenic mice received 104 OT-I CD8+ T cells prior to infection with 2×10^7^ PINK amastigotes. From day 15 pi on mice were treated biweekly with anti-B7-H1 antibodies. Animals were sacrificed at indicated times pi. (A) Modulation of CD62L and CD122 expression on OT-I CD8+ T cells, identified by gating on Ly5.2+ CD8+ cells. Mean percentages of cells expressing low/intermediate levels of CD62L (left panel) and CD122 (right panel) are shown. (B) Splenocytes from ant-B7-H1 treated and isotype control treated mice were restimulated in vitro for 4 h with the SIINFEKL peptide and IFNg and TNFa were assessed by ICS. Graphs represent the percentage of OT-I CD8+ T cells producing IFNg (left panel) and TNFa (right panel). All data represent mean±se, n = 3. (C) C57BL/6 mice were infected with 2×10^7^ LV9 amastigotes and treated biweekly from day 15 pi on with anti-B7-H1 antibodies. Mice were sacrificed at indicated time after infection. Graph represents the hepatic parasite burden expressed as LDU. All data represent mean±se of one experiment, n = 5. (0.28 MB TIF) [file ppat.1000431.s006.tif]

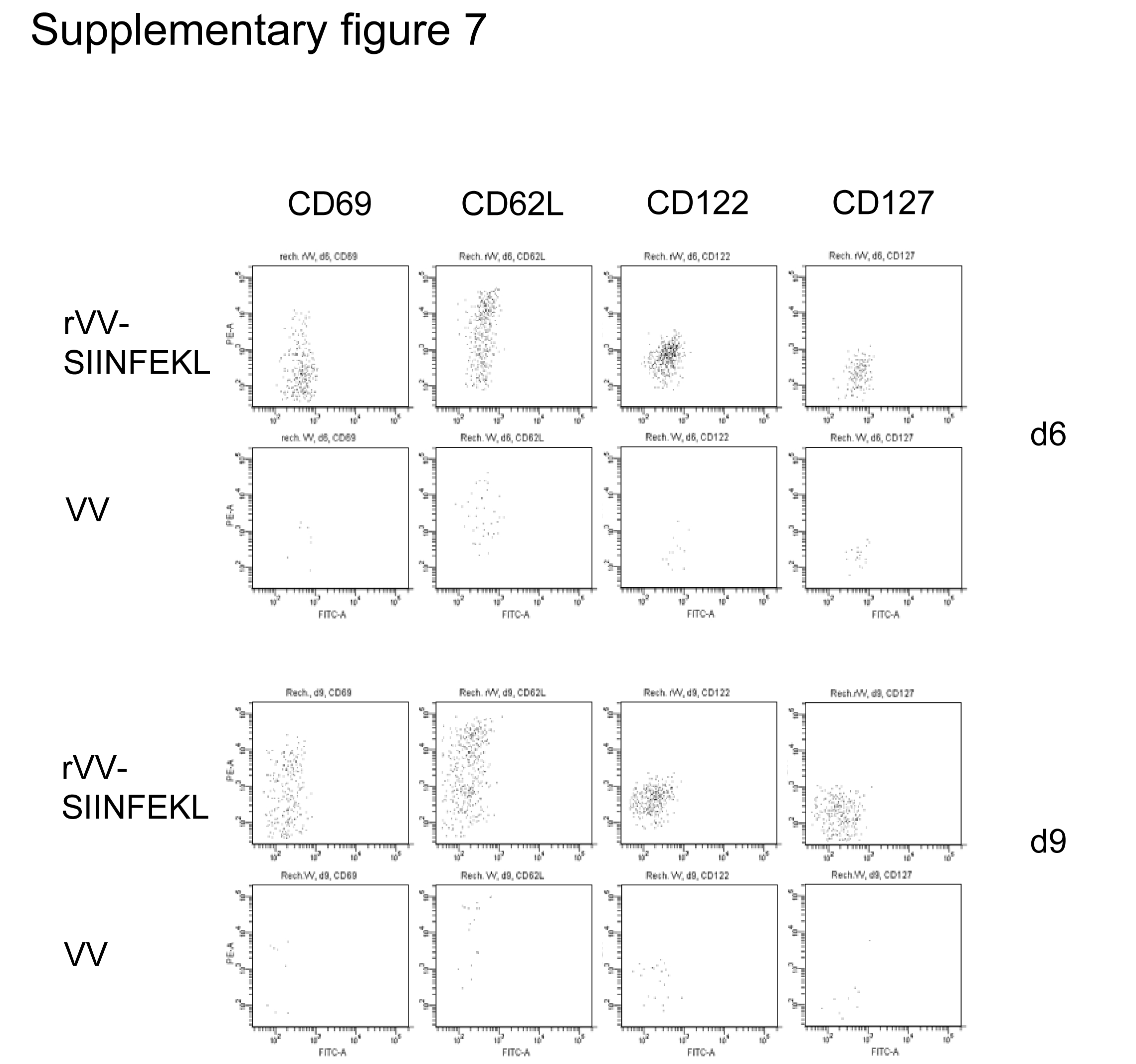

Supplement: Figure S7 — Modulation of expression of CD62L, CD69, CD122, and CD127 after superinfection with VV and/or rVV-SIINFEKL. Representative plots for both groups at day 6 and 9 after challenge are shown. (0.47 MB TIF) [file ppat.1000431.s007.tif]
